# Measuring functional connectivity in MEG: A multivariate approach insensitive to linear source leakage

**DOI:** 10.1016/j.neuroimage.2012.03.048

**Published:** 2012-11-01

**Authors:** M.J. Brookes, M.W. Woolrich, G.R. Barnes

**Affiliations:** a Sir Peter Mansfield Magnetic Resonance Centre, School of Physics and Astronomy, University of Nottingham, University Park, Nottingham, UK; b Oxford Centre for Human Brain Activity, University of Oxford, Warneford Hospital, Oxford, UK; c Wellcome Trust Centre for Neuroimaging, University College London, London, UK

**Keywords:** MEG, Neural oscillations, Functional connectivity, Multivariate analysis

## Abstract

A number of recent studies have begun to show the promise of magnetoencephalography (MEG) as a means to non-invasively measure functional connectivity within distributed networks in the human brain. However, a number of problems with the methodology still remain — the biggest of these being how to deal with the non-independence of voxels in source space, often termed signal leakage. In this paper we demonstrate a method by which non-zero lag cortico-cortical interactions between the power envelopes of neural oscillatory processes can be reliably identified within a multivariate statistical framework. The method is spatially unbiased, moderately conservative in false positive rate and removes linear signal leakage between seed and target voxels. We demonstrate this methodology in simulation and in real MEG data. The multivariate method offers a powerful means to capture the high dimensionality and rich information content of MEG signals in a single imaging statistic. Given a significant interaction between two areas, we go on to show how classical statistical tests can be used to quantify the importance of the data features driving the interaction.

## Highlights

► We demonstrate a method to image cortico-cortical interactions in MEG data. ► The method employs a multivariate statistical framework in MEG source space. ► The technique has reduced sensitivity to signal leakage across voxels. ► In simulation we show spatial accuracy and slightly conservative false positive rate. ► In real data we show cortico-cortical interaction between left and right motor cortex.

## Introduction

In recent years, neuroscience and neuroimaging have been revolutionised by the discovery of large distributed brain networks, some associated with simple sensory processing (e.g. the motor network) and others associated with higher level function (e.g. the default mode network). Functional magnetic resonance imaging (fMRI) has become a modality of choice for characterisation of functional connectivity within these networks, both at rest and during tasks (see e.g. Smith et al., 2009). However, fMRI is an indirect measure of brain activity based on haemodynamic responses to underlying electrodynamics. Its indirect nature and limited temporal resolution mean that fMRI cannot probe the electrical processes that mediate connectivity or the most rapid temporal fluctuations in network activity. Magnetoencephalography (MEG) detects extra-cranial magnetic fields induced by synchronised neuronal current flow in the brain. In this way it offers advantages over indirect techniques such as fMRI as it can bypass haemodynamics and probe phenomena such as neural oscillations which are thought to be a more direct manifestation of cortical connectivity (Schnitzler and Gross, 2005; Singer, 1999). The utility of MEG (and electroencephalography (EEG)) to measure connectivity has been shown by a large number of studies (Gow et al., 2008; Gross et al., 2001, 2002; Ioannides et al., 2000; Jerbi et al., 2007; Nolte et al., 2004, 2008; Ramnania et al., 2004; Schlögl and Supp, 2006; Schoffelen and Gross, 2009; Tass et al., 1998). Recent work has also begun to show that networks observable in fMRI can also be detected using MEG (Brookes et al., 2011a,b; de Pasquale et al., 2010; Liu et al., 2010). These later studies have measured temporal correlation between power envelopes of neural oscillatory signals at separate brain space voxels. In this way, network maps of correlated signals have been generated and shown to match spatially network maps in fMRI, providing electrophysiological validation of a number of fMRI resting state networks. MEG however has the potential to move beyond fMRI, and elucidate the electrodynamic processes that underpin network formation, without confounds associated with haemodynamic responses.

Despite the promise of MEG as a means to measure connectivity, a number of methodological difficulties remain (Schoffelen and Gross, 2009). Using source space projected measurements, the principal limitation is that signals reconstructed at spatially separate brain locations are not necessarily independent; an artifact of the ill posed MEG inverse problem (reconstructing brain space current density measures from scalp based magnetic recordings). This non-independence means that spurious connectivity can exist between two projected MEG time series that is due entirely to signal leakage between projections. A number of techniques to correct for this leakage have been tried; one of the most successful approaches being the use of the imaginary parts of the linear interaction to quantify coherence (Guggisberg et al., 2008; Nolte et al., 2004). However, coherence assumes that signals from two connected brain areas have a fixed phase lag between them, which is not necessarily the case, even for short time windows. Recent studies using envelope correlation methods (Brookes et al., 2011a) have presented techniques to test for leakage via simulation, however such methods are highly computationally intensive and, whilst offering a means to identify spurious connectivity, do not offer a means to eliminate it. A further difficulty in MEG connectivity measurement lies in statistical thresholding of connectivity maps. Using a seed based correlation approach, Pearson correlation between the signal from a seed voxel, and signals from all other voxels is computed. Whilst these correlation values can be converted to statistical quantities (assuming accurate knowledge of temporal degrees of freedom) correction for multiple comparisons across voxels is non trivial due to the inherent spatial smoothness and inhomogeneity in MEG source space images. Finally, the richness of MEG data means that connectivity maps are generally constructed using data that have been frequency filtered into multiple frequency bands of interest (classical alpha, beta and gamma bands for example). This means that a single experiment yields multiple connectivity maps, making results difficult to interpret and leading to a further multiple comparison problem.

In this paper, we address some of the limitations currently associated with MEG source space functional connectivity analysis by application of a multivariate statistical framework. Previous work has shown the utility of a multivariate approach both for identification of activity in brain areas elicited by a task (Barnes et al., 2011; Soto et al., 2009) and in connectivity analysis (Soto et al., 2010). In Soto et al. the authors put forward a scheme which eliminates leakage by suppressing any interactions in which the two (canonical) vectors describing power–power coupling between two regions are co-linear. In this work, we employ a similar, but less conservative, multivariate framework to investigate power–power coupling within or between frequency bands, across spatially separate brain areas. Here we address the signal leakage problem by regressing out the linear projection of the seed voxel from the signals at the test voxel. We subsequently assess power–power coupling both within and across multiple frequency bands, using a multivariate statistical framework and a previously described technique (Barnes et al., 2011) to correct for multiple comparisons across voxels. Simulation results show that our statistical analyses yield slightly conservative estimates of false positive rate, invariant of source space, or the number of features used in the connectivity analysis. We also apply our approach to real resting state MEG data showing that our method can identify significant cross hemisphere power–power coupling in the motor cortices.

## Theory

Our method, comprises the following steps: 1) source space projection of MEG data using beamforming; 2) correction for signal leakage effects by regressing out (orthogonalising with respect to) a linear projection of the seed voxel time course; 3) multivariate statistical analysis of interdependencies between timecourses; 4) correction for multiple comparisons across voxels; and 5) further quantification of the nature of the significant interaction. These five steps are described in detail below.

### Beamforming

Using a beamformer (Barnes et al., 2011; Gross et al., 2001; Robinson and Vrba, 1998; Van Drongelen et al., 1996; Van Veen et al., 1997), an estimate of electrical source strength ${\hat{Q}}_{\psi }\left(t\right)$, is made at time *t* and at a predetermined location in the brain using a weighted sum of MEG sensor measurements:

(1) $${\hat{Q}}_{\psi }\left(t\right)=W_{\psi }^{T}m\left(t\right)\text{.}$$

Here, ***m***(*t*) is a (*N*_sens_ × 1) vector of magnetic field measurements made at time *t*, and ***W***_**ψ**_ is a (*N*_sens_ × 1) vector of weighting parameters tuned to a specific source space location and current orientation (*N*_sens_ represents the number of MEG sensors). Location and orientation are represented by **ψ**. Superscript *T* indicates a matrix transpose. The weighting parameters (***W***_**ψ**_) are derived based on power minimisation. The overall power in ${\hat{Q}}_{\psi }\left(t\right)$ is minimised with a linear constraint that power originating from location/orientation **ψ** remains. A solution to this problem is (Van Veen et al., 1997):

(2) $$W_{\psi }^{T}={\left[L_{\psi }^{T}{\left\{C+\mu \Sigma \right\}}^{-1}L_{\psi }\right]}^{-1}L_{\psi }^{T}{\left\{C+\mu \Sigma \right\}}^{-1}$$

where **C** represents the data covariance matrix calculated over a time–frequency window of interest and ***L***_**ψ**_ is the lead field vector (containing the magnetic fields that would be measured at each of the MEG sensors in response to a dipole source of unit amplitude with location and orientation **ψ**). **Σ** is a diagonal matrix representing the white noise at each of the MEG channels and *μ* is a Backus–Gilbert regularisation parameter (here we use *μ* = 2) (Brookes et al., 2008). Time series ${\hat{Q}}_{\psi }\left(t\right)$ are reconstructed for a set of locations placed at the vertices of a regular (5 mm in this case) grid spanning the entire brain. The orientation of each source was based on a non-linear search for the orientation of maximum signal to noise ratio (Robinson and Vrba, 1998). Following projection of MEG data via beamformer, our subsequent aim is to measure connectivity via assessment of interactions between projected signals at two separate locations, which we shall refer to as the ‘seed’ location (**ψ**_S_) and the ‘test’ location (**ψ**_T_). The projected MEG data from these two locations (${\hat{Q}}_{\psi_{\text{S}}}\left(t\right)$ and ${\hat{Q}}_{\psi_{\text{T}}}\left(t\right)$) are segmented into *N* time blocks of equal length Δ. Each block is then Fourier transformed to yield *fΔ* complex valued Fourier coefficients and thus two matrices **X** and **Y** are formed, both of dimension *N* × *fΔ*, where **X** represents data from the seed location and **Y** represents data from the test location. *f* represents half the MEG sampling frequency.

### Signal leakage correction

To remove the effect of signal leakage between **X** and **Y** these matrices are initially reshaped into 1D vectors, **x** and **y**, containing the concatenated Fourier coefficients from all frequency elements across all time blocks. In the beamformer formulation employed here, the same weighting parameters (i.e. the same spatial filters) are employed for all frequencies and so signal leakage between voxels is expected to affect all frequency components equally. To remove the effect of signal leakage, a univariate projection of the vector **x** on **y** is estimated:

(3) $$\beta_{\mathrm{UV}}=x^{+}y$$

where **x**^+^ denotes the pseudo-inverse of **x**. [Note that whilst in theory **x**^+^**y** is always real, numerical issues can introduce a finite imaginary component; in such cases Eq. (3) is replaced by $\beta_{\mathrm{UV}}=Re\left(x^{+}y\right)$ where Re denotes the real component of **x**^+^**y**.] The linear projection (or estimate of **y** based on vector **x**) is removed thus:

(4) $$y_{R}=y-x\beta_{\mathrm{UV}}$$

where **y**_**R**_ is the component of **y** that is orthogonal to **x**. In this way any linear interaction between **x** and **y** is removed. Since signal leakage will always give rise to a zero phase lag linear interaction, this step enables reduction of signal leakage (at the expense of true zero lag interactions, see Discussion). Note that projection is carried out here in time–frequency space. However, the same operation could be performed on the original time series (${\hat{Q}}_{\psi_{\text{S}}}\left(t\right)$ and ${\hat{Q}}_{\psi_{\text{T}}}\left(t\right)$). These two techniques are equivalent: Performing correction on raw time series is intuitive; however it would necessitate frequency filtering of raw data to the band of interest prior to correction; projection in time frequency space enables simple exclusion of frequency components of no interest. A univariate predictor, *β*_*UV*_, of the linear interaction is derived here, meaning that a single number must represent linear interactions across all frequency bands. This is based on the assumption that signal leakage between the seed and test locations will be equivalent across all frequency components. However, given sufficient degrees of freedom, it would be possible to extend this methodology and perform correction separately on a frequency band specific basis if deemed necessary.

It is important to note at this stage that if the seed and test locations coincide (i.e. **Y** = **X**) Eq. (4) will result in zero signal and imaging statistics described below may be ill defined. To avoid this, throughout this paper, seed locations (**ψ**_S_) are displaced from the test locations (**ψ**_T_) by 0.1 mm when computing the imaging statistics.

### Multivariate test

Following signal leakage correction, **x** and **y**_**R**_ are again reshaped into *N* × *fΔ* matrices **X** and **Y**_**R**_ respectively. The absolute value of the Fourier coefficients are computed, and the columns of **X** and **Y**_**R**_ are collapsed across frequency elements to yield two new matrices, **X**_**P**_ and **Y**_**PR**_ in which each column represents the (mean corrected) oscillatory power contained in a single frequency band (see Methods: experimental data for a list of the bands used), whilst rows represent separate time blocks (in this case duration Δ = 1 s). The dimensions of both **X**_**P**_ and **Y**_**PR**_ are thus *N* × *N*_B_ where *N*_B_ is the number of frequency bands.

This preprocessing leaves the confound that the columns of **X**_**P**_ and **Y**_**PR**_ could be correlated, particularly if frequency bands are adjacent to one another. It is much simpler to deal with orthogonal columns and so (for the real data presented below where the source spectra are unknown a-priori) the columns of **X**_**P**_ and **Y**_**PR**_ are orthogonalised. This is achieved by first computing the covariance matrices **C**_**XX**_ = **X**_**P**_^*T*^**X**_**P**_ and **C**_**YY**_ = **Y**_**PR**_^*T*^**Y**_**PR**_. Eigenvalue decomposition of these matrices then yields a set of eigenvalues, and eigenvectors. These eigenvalues are truncated, retaining those that explain 99% of the total variance in the data and a new, orthogonalised version of **X**_**P**_ and **Y**_**PR**_ is created as **X**_**o**_ = **X**_**P**_**U**_**X**_ and **Y**_**o**_ = **Y**_**PR**_**U**_**Y**_ where the columns of **U**_**X**_ and **U**_**Y**_ represent the eigenvectors of **C**_**XX**_ and **C**_**YY**_ respectively. In the remainder of this manuscript the term *feature* is used to describe a column of **X**_**o**_ (i.e. a feature is a linear combination of frequency bands). The dimensions of both **X**_**o**_ and **Y**_**o**_ are *N* × *N*_F_ and *N*_F_ (≤ *N*_B_) is the number of features. [Note that in the case of simulated data, orthogonalisation is not necessary; in these special cases the number of features is equal to the number of frequency bands.]

Following orthogonalisation, power fluctuations at the test location (**Y**_**o**_) are represented by a general linear model, where the design matrix (**X**_**o**_) contains power fluctuations at the seed location:

(5) $$Y_{o}=X_{o}\beta +\varepsilon \text{.}$$

The maximum likelihood prediction of **Y**_**o**_ based on **X**_**o**_ (assuming Gaussian distributed error, **ε**) is given by:

(6) $$T=X_{o}\beta$$

(7) $$\beta =X_{o}^{+}Y_{o}\text{.}$$

The multivariate test procedure (also described in Barnes et al., 2011) then involves testing the following expression of the null hypothesis:

(8) $$\left|H-\theta R\right|=0$$

where **H** represents the covariance explained by the least squares prediction of **Y**_**o**_:

(9) $$H=T^{T}T$$

and **R** is the unexplained covariance:

(10) $$R={\left(Y_{o}-T\right)}^{T}\left(Y_{o}-T\right)\text{.}$$

In the simplest case, if **X**_**o**_ and **Y**_**o**_ comprised a single column, the single eigenvalue is given by θ=HR, i.e. the variance explained divided by the variance of the residuals, or the univariate F statistic. In the more general multivariate case the Wilk's Lambda statistic can be expressed as a function of the eigenvalues *θ*_*i*_ (with corresponding eigenvectors **a**_*i*_) of the matrix **R**^− 1^**H**:

(11) $$\Lambda =\prod_{i=1}^{s}\frac{1}{1+\theta_{i}}:s=\min\left(\nu ,h\right)$$

where *ν* and *h* are the ranks of (number of columns in) **Y**_**o**_ and **X**_**o**_ respectively. With appropriate transformation (Chatfield and Collins, 1980) and for large degrees of freedom, this can be approximated to a *χ*^2^ statistical distribution.

(12) $$-\left(r-\frac{\nu -h+1}{2}\right)\ln\left(\Lambda \right)\sim \chi^{2}\left(\nu h\right)$$

where *r* = *N* − *ν* − *h*. This statistic can be computed on a voxel by voxel basis to test for linear mixtures (given by **a**_*i*_) of columns in **Y**_**o**_ that co-vary with linear mixture (given by **b**_**i**_, see later) of columns in **X**_**o**_ at the seed location. Note the significant advantages here over previous correlation based methods that: 1) effects of signal leakage have been removed from the data via the linear subtraction described by Eq. (4); and 2) effects of interest across all frequency bands have been collapsed into a single statistic.

### Multiple comparison correction

We use the heuristic introduced by Barnes et al. (2011) to correct for multiple comparisons across voxels within the multivariate statistical framework described above. Briefly, if **L** represents the lead field matrix containing a single row for each of the *p* voxels in source space, then we construct a new matrix with *p* rows and two columns containing the 2 channel indices of the maximum and minimum field measurement in each row; i.e. these indices correspond to the field map peaks for any lead field. The estimate of the number of independent elements *ρ* is then given by the number of unique rows (regardless of order of maxima and minima). Statistical thresholding of the *χ*^2^ image, derived above, is then corrected by dividing the required family wise error rate (*α*_FWE_) by *ρ* to give a corrected threshold *α*_corr_ for the volume.

(13) $$\alpha_{\text{corr}}=\frac{\alpha_{\text{FWE}}}{\rho }$$

### Testing the most significant features

The multivariate statistical test described above is used to identify if there is a significant interaction between the seed location and any other brain region. We now look to quantify the dominant features (i.e. linear combination of frequency bands) driving this interaction. However, there may be more than one eigenmode (each characterised by a single eigenvector, **a**_i_, and associated eigenvalues *θ*_*i*_) describing a significant interaction, and we wish to find the dominant features for each one. The first thing we do is to test for the number of significant eigenmodes, as follows: if *d* = 0 corresponds to the dominant mode in Eq. (11) above then the probability that there are subsequent significant components at modes *d* = 1,2, etc. is given by:

(14) $$\left(r-\frac{\nu -h+1}{2}\right)\ln\prod_{i=1+d}^{s}\left(1+\theta_{i}\right)\sim \chi^{2}\left(\left(\nu -d\right)\left(h-d\right)\right)\text{.}$$

That is, it is possible to reduce a significant interaction into a number of significant eigenmodes. The eigenvalue *θ*_*i*_ of each mode determines its significance and the eigenvector **a**_*i*_ determines the linear combination of features that maps **Y**_**o**_ to **y**_*i*_ '. Whilst correspondingly, the projection of **X**_**o**_ onto **x**_*i*_ ' is given by **b**_*i*_:

(15) $$b_{i}=\beta a_{i}$$

(16) xi'=Xobi

(17) yi'=Yoai

where *i* indexes the eigenmode in question.

Now that we know the number of significant eigenmodes, we can also determine which features are driving the interaction within each mode. For example, let us assume that only the first eigenmode was significant (*d* = 0 in Eq. (14)) then the model describing the interaction is

(18) $$\left(r-\frac{\nu -h+1}{2}\right)\ln\prod_{i=1}^{s}\left(1+\theta_{i}\right)\sim \chi^{2}(vh)\text{.}$$

We now look to trim away the columns of **X**_**o**_ leaving only those essential to the interaction. The orthogonalisation of the features in **Y**_**o**_ and **X**_**o**_ is useful here, as it provides a simple hierarchy of testable models. We begin with the column that explains most of the variance (*h* = 1) and then progressively add columns. The difference between two *χ*^2^ distributions is also *χ*^2^ (distributed as the difference in the two degrees of freedom). So in order to compare two models, with different numbers of columns Δ_*h*_, the null distribution of the difference between models (where **Y**_**o**_ remains of rank *v*) is distributed.

(19) $$\chi^{2}(v(h+\Delta_{h}))-\chi^{2}(vh)\sim \chi^{2}(v\Delta_{h})$$

Note, to simplify notation, for the remainder of this manuscript we use **X** to denote **X**_**o**_ and **Y** to denote **Y**_**o**_.

## Methods: simulations

All simulations were undertaken using MEG system geometry based on the third order synthetic gradiometer configuration of a 275 channel whole head CTF MEG system. The location of the brain anatomy with respect to the sensors was taken from a real experimental recording. The lead fields for all simulated dipolar sources were based on a multi-sphere head model (Huang et al., 1999) and the dipole equations described by Sarvas (1987). Additive noise data were generated by experimental recording: ten 300 s MEG recordings were made using the third order synthetic gradiometer configuration of a 275 channel CTF MEG system at a sampling rate of 600 Hz, with no subject in the scanner. These ‘empty room’ recordings were concatenated yielding 3000 s of noise data, epochs from which could be randomly selected and added to simulated MEG data. It is noteworthy that previous simulation work shows a marked difference between simulated noise (Gaussian random noise uncorrelated across sensors) and real measured noise (which is not Gaussian in nature and is correlated across sensors due to external environmental magnetic interference) (Brookes et al., 2010). We surmised that the use of real noise would represent a more realistic test of the methodology.

### Proof of principle

Two dipolar sources were simulated, located in the left and right primary motor cortices. The source timecourses comprised Gaussian random noise frequency filtered into the 1–150 Hz band with amplitude 5 nAm. The source orientations were tangential to the radial orientation but randomised with respect to the azimuthal direction. A ‘power–power’ interaction was simulated between the sources via addition of an ‘interaction’ signal; in the form of a co-modulation of the two source envelopes. The interaction signal was generated by filtering Gaussian random noise into the 20–40 Hz frequency band and multiplying the resulting signal by a 0.1 Hz sinusoid (amplitude 5 nAm). Although the two sources share the same envelope modulation, the underlying time-courses (generated from filtered noise) were uncorrelated. In this way the two source timecourses mimicked power–power interdependencies of the type previously shown to exist in real data (Brookes et al., 2011a,b; de Pasquale et al., 2010; Liu et al., 2010). The interaction signals were added to the basic broadband source timecourses and multiplied by a dipolar lead field to generate simulated MEG data. Empty room noise data were then added to the simulated source data. These data were processed using the technique outlined in the theory section, with the seed location taken to be the simulated source in the right motor region. *χ*^2^ images depicting simulated connectivity were reconstructed on a 5 mm cubic grid spanning the entire brain. This process was repeated with and without signal leakage correction (in the case without signal leakage correction **y**_**R**_ = **y**).

### Testing false positive rates

In order to test the multivariate statistical approach, along with our approach to eliminating linear interactions, we ran four sets of simulations each containing multiple noise realisations. In all cases sources were simulated as above but with no simulated interactions. Source timecourses comprised Gaussian random noise frequency filtered into the 1–150 Hz band with amplitude 5 nAm. The source orientations were tangential to the radial orientation but randomised with respect to the azimuthal direction.

1) A single source was simulated in the right primary motor area. The source timecourse was constructed as above, and empty room noise data were added. *χ*^2^ images were constructed taking the seed location to be that of the simulated source and using voxels spaced 1 cm apart on a regular cubic grid spanning the entire brain. 500 realisations of this simulation were run, with both the simulated source timecourse, and the empty room noise, changing for each realisation. Signal leakage correction was applied in all cases.
2) Two sources were simulated in the left and right primary motor areas. Both source timecourses were constructed as above but unlike the case for our proof of principle simulation, no interaction between sources was simulated meaning that any connectivity identified in the resulting statistical images would be entirely artifactual. Empty room noise data were then added. The multivariate analysis was applied taking the seed location to be that of the simulated source in the right motor region and *χ*^2^ images were constructed on a 1 cm grid. 500 realisations of this simulation were run, with both simulated source timecourses and empty room noise changing for each realisation. Signal leakage correction was applied in all cases.
3) Identical to simulation 1, but with statistical images constructed on a regular 2 cm cubic grid spanning brain space and the number of realisations increased to 4000.
4) The number of features was altered between 1, 2, 3, 5 and 7 by changing the number of frequency bands studied. In each case, 250 realisations of the simulation were run, with images reconstructed on a 2 cm grid spanning the whole brain.

In all simulations, a single false positive was defined as a realisation of the simulation in which one or more voxels was significant at (p < *α*_corr_). We computed this empirical rate for a range of values of the desired family wise error rate *α*_FWE_. The locations of the local maxima within these *χ*^2^ images were also recorded and further analysed.

## Methods: experimental data

### Data collection

In order to test the method experimentally, previously described MEG data (Brookes et al., 2011a) were used. MEG data were recorded using the third order gradiometer configuration of a 275 channel CTF MEG system at a sampling rate of 600 Hz. The scanner is housed inside a magnetically shielded room (MSR) and a 150 Hz low pass anti-aliasing hardware filter was applied. The study was approved by the University of Nottingham Medical School Research Ethics Committee and data from a single subject are considered here. The recording comprised two phases; in the first phase, 300 s of resting state MEG data were acquired during which the subject was asked to remain awake with their eyes open and fixate on a marker, which was displayed on a screen located approximately 40 cm in front of the subject. In the second phase of the experiment, the subject undertook a motor task (see Brookes et al., 2011a for details). Note that in this paper, the motor task was used only to identify a seed location in the primary motor area. The data acquired during the 300 s resting state phase of the experiment were processed using our multivariate method in order to search for left–right motor cortex resting state connectivity. Left–right motor cortex power–power coupling has been demonstrated in previous papers (Brookes et al., 2011a,b).

During data acquisition the location of the subject's head within the scanner was measured by energising coils placed at 3 fiducial points on the head (nasion, left preauricular and right preauricular). Following data acquisition, the positions of the coils were measured relative to the subject's head shape using a 3D digitiser (*Polhemus isotrack*). An MPRAGE structural MR image was acquired using a Philips Achieva 3T MRI system (1 mm^3^ isotropic resolution, 256 × 256 × 160 matrix size). The locations of the fiducial markers and MEG sensors with respect to the brain anatomy were determined by matching the digitised head surface to the head surface extracted from the anatomical MRI.

### Data analysis

Data were mean corrected on a trial by trial basis and frequency filtered to the 1–150 Hz range. Periods of data containing large artifacts were identified and discarded. A seed location was defined as previously described for these same data (Brookes et al., 2011a) using the motor task and applying Synthetic Aperture Magnetometry (SAM) (Robinson and Vrba, 1998). This method has been shown to give accurate spatial measurements of the motor areas (Gaetz et al., 2011; Stevenson et al., 2011). A seed location was chosen to be at the local image maximum within the right primary motor area.

Having identified a seed location, multivariate analysis was applied to resting state data, as described above, using a broad frequency range (4–80 Hz). MEG data were projected into source space using the beamformer technique with covariance based on the 300 s resting state data only. Data were then segmented into 1 s time windows, Fourier transformed within each segment and divided into 9 frequency bands (4–8 Hz; 8–13 Hz; 13–20 Hz; 20–30 Hz; 30–40 Hz; 40–50 Hz; 50–60 Hz; 60–70 Hz; 70–80 Hz) and subsequently orthogonalised into 5 modes explaining 99% of the variance. A *χ*^2^ connectivity map was computed with and without correction for signal leakage, the difference image ([*corrected*] − [*uncorrected*]) was also computed. This multivariate analysis was repeated twice, once with real resting state data, and a second time where the beamformer spatial filters were derived based on real data, but data were replaced with a five minute empty room recording prior to subsequent application of multivariate analysis. This latter image computation would highlight any spatial structure in the connectivity image that resulted solely from beamformer inverse solution. An image of correlation between beamformer weights at the seed location, and all other locations in the head was also derived. All final connectivity images were thresholded at *α*_FWE_ < 0.05 (i.e. corrected across the image volume for multiple comparisons) using the lead-field based metric described above.

Having identified a significant interaction between the seed and another region we sought to quantify the dominant features (frequency band) driving this interaction. This began with a test for the number of significant eigenmodes (Eq. (14)). For each significant (*α* < 0.05) mode we performed a hierarchical test on models containing increasing numbers of orthogonal features in **X**.

## Results: simulations

**Fig. 1:**
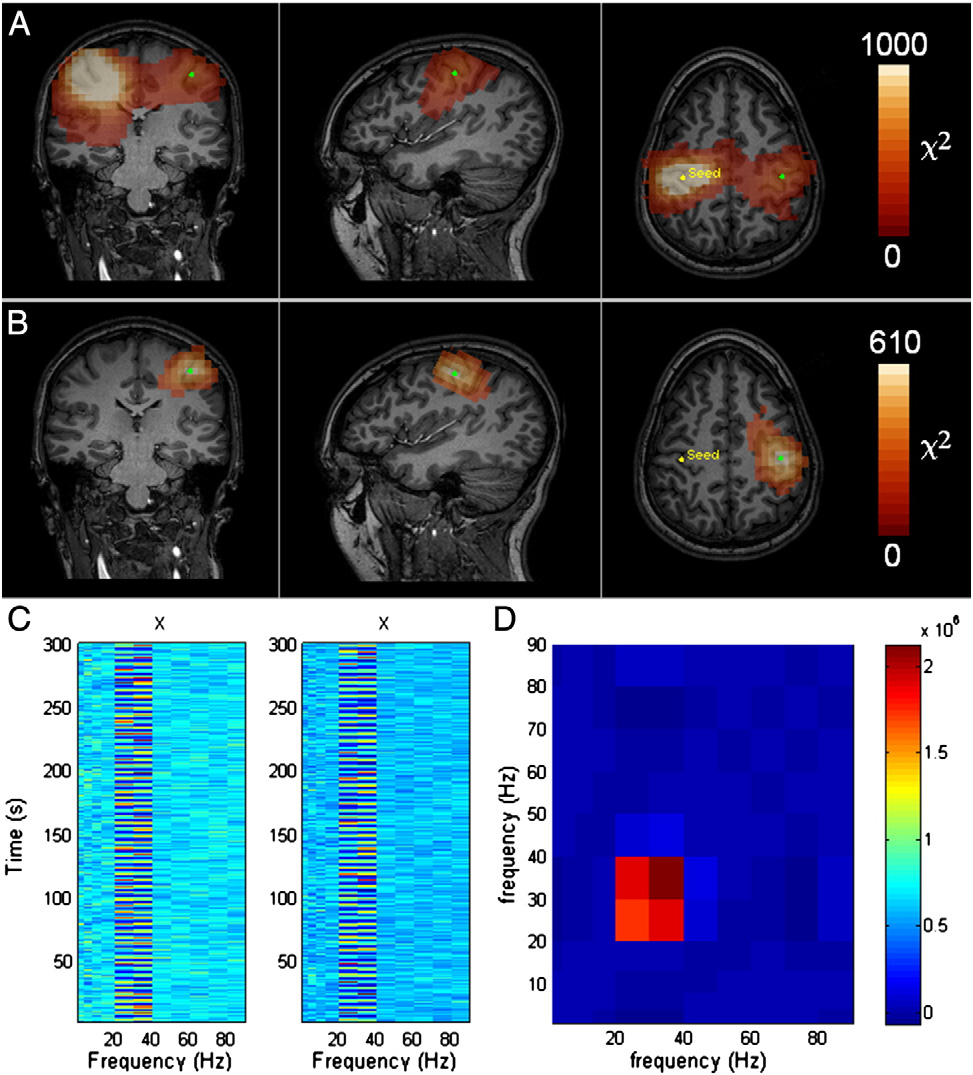
Two interacting sources: Seed based analysis using a multivariate approach. The seed is in the right hemisphere (left hand side of the image as shown in yellow). The interacting source is in the left hemisphere at the location marked by the green dot. Panel A shows multivariate analysis without subtraction of linear interactions. [Note, this has been windowed to show the interaction; a large peak is observed precisely at the seed location which is not visible here due to windowing]. B) The equivalent case with subtraction of linear interaction. Note that seed blur has been eliminated completely. Note also that in both cases the interacting source is successfully located. C) A visualisation of **X** and **Y**, extracted from the two simulated source locations and showing sinusoidal interactions in the 20–40 Hz band. D) Visualisation of H, the variance explained as a function of frequency, showing interaction between the simulated sources in the 20–40 Hz band as expected.

Fig. 1 shows the results of our proof of principle simulation. Fig. 1A shows the *χ*^2^ images for simulated MEG data with two interacting sources in the left and right motor regions. In this case we have not corrected for signal leakage and the seed location is in the right motor area. As shown, the multivariate approach successfully locates the interacting source which has been placed in the left motor cortex. Also apparent is a large area in the right hemisphere, distributed asymmetrically around the seed location, which is an example of seed blur. Fig. 1B, shows an equivalent functional connectivity map, but in this case, correction for signal leakage has been applied. Notice that the second interacting source in the left hemisphere remains accurately localised, but seed blur is eliminated completely. In both cases, the images have been thresholded at *α*_FWE_ < 0.01. Fig. 1C shows a visualisation of **X** and **Y** (not orthogonalised), extracted from simulated source locations in the left and right motor areas. This shows graphically the sinusoidal interaction simulated between the two sources in the 20–40 Hz band. Finally, Fig. 1D shows **H**, the variance explained as a function of frequency (note again that for this visualisation, **X** and **Y** were not orthogonalised meaning that **H** is genuinely representative of the raw frequency bands). Here the expected interaction in the 20–40 Hz band is highlighted. This figure exemplifies the power of the approach described; data have been analysed across a broad frequency range (comprising 7 distinct bands), with interactions simulated in the 20–40 Hz band only. In addition to accurate localisation of the interacting source, the method also effectively eliminates seed blur.

**Fig. 2:**
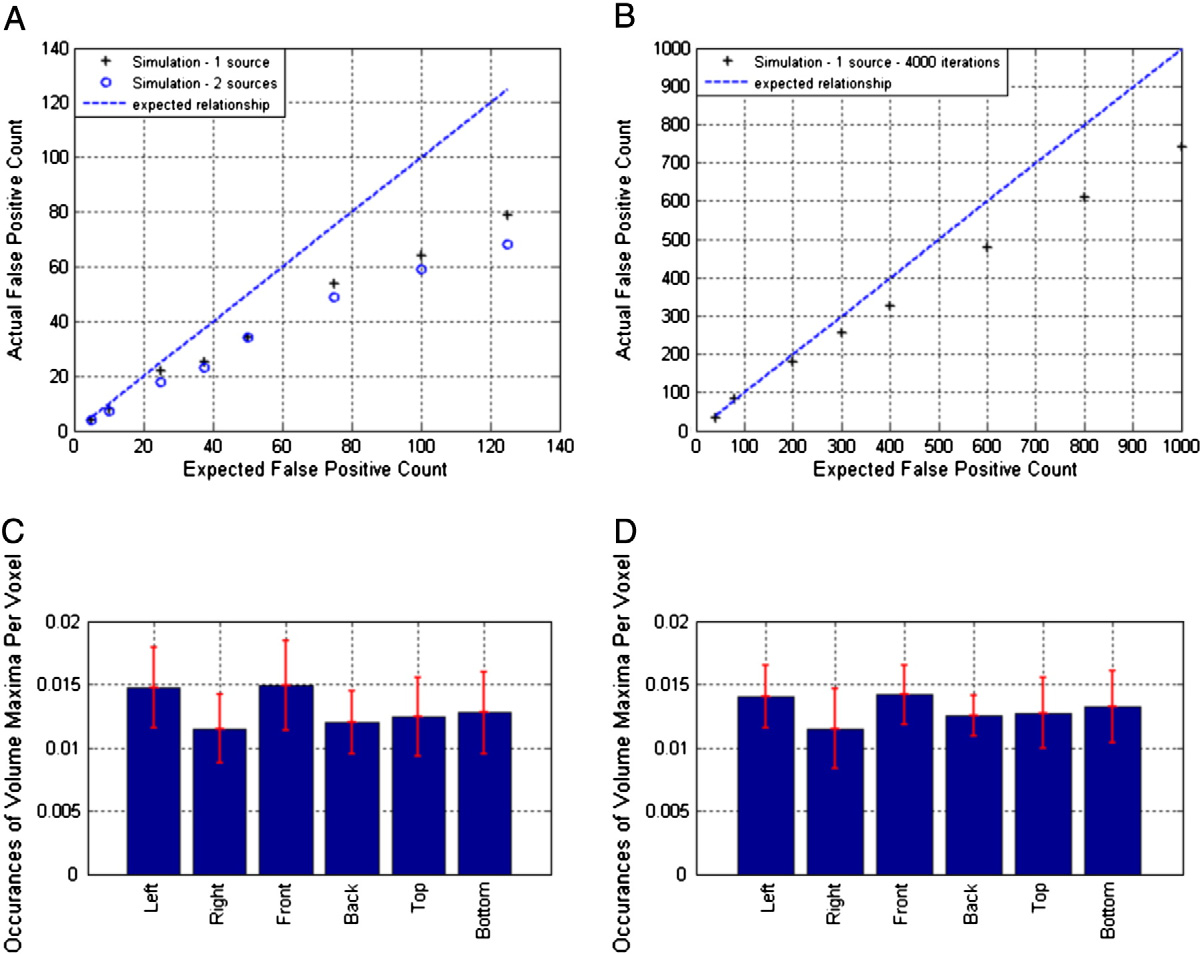
Result of testing for false positive rate in simulated data. Panel A shows the actual false positive rate (generated from the simulation) plotted against the expected false positive rate. Crosses show the case for simulations with 1 source. Circles show the case for 2 simulated sources with no interaction. Panel B shows the false positive rate for a simulation in which connectivity images are reconstructed on a 2 cm grid and 4000 realisations are performed. C) The spatial locations of maxima in 500 realisations of the two source simulation. D) The spatial locations of maxima in 500 realisations of the single source simulation.

Fig. 2 shows the achieved vs. expected false positive rate counts for simulated data (simulations 1, 2 and 3 above). Fig. 2A shows the actual false positive rate plotted against the expected false positive rate for a single simulated source (simulation 1 ‘+’) and two non-interacting simulated sources (simulation 2 ‘o’). 500 realisations of both simulations were run, and for each realisation the source timecourse, source orientation and the noise data were randomised. Connectivity images were reconstructed on a 1 cm grid spanning the whole brain. For 7 features in both **X** and **Y** the null is distributed *χ*^2^(*νh* = 49). Examining the lead field structure gave *ρ* = 1164 and so for *α*_FWE_ < 0.01 we looked for voxels significant at p < 0.01/1164. If any of the image voxels in a single realisation exceeded the threshold the false positive count was incremented. Note good agreement between actual and expected false positive counts with statistics becoming conservative for larger values p values. Fig. 2B shows an equivalent result where connectivity images are reconstructed on a 2 cm grid and 4000 realisations of the single source simulation are performed. Again note agreement with the expected false positive rate. To test if the choice of a seed location and removal of its linear projection caused a spatial bias of the statistical images, Figs. 2C/D show the distribution of spatial locations of the local maxima in the 500 connectivity maps computed for simulation 1. Fig. 2C shows the case for a two source simulation (i.e. simulation 2) whilst Fig. 2D shows the case for a single source simulation (i.e. simulation 1). To generate these bar charts, the total number of image maxima appearing in the left/right hemispheres, anterior/posterior hemispheres and upper/lower hemispheres has been computed, and normalised by the number of voxels in each of those regions. Results show that the locations of image local maxima are approximately evenly distributed; i.e. false positives are not biased towards seed locations.

**Fig. 3:**
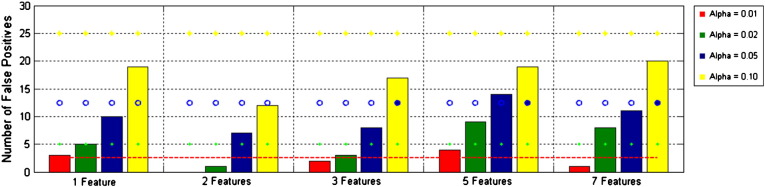
Further simulation results: The number of false positives achieved (out of 250) for different values of *α*_FWE_ (indexed by colour) over tests with different numbers of features. Ideal rates are given by dashed lines of the same colours.

Fig. 3 shows the number of false positives as a function of p-value, for 5 different numbers of features (i.e. numbers of frequency bands considered). It is noteworthy that the false positive count appears independent of the number of features used in the multivariate analysis.

## Results: experimental data

**Fig. 4:**
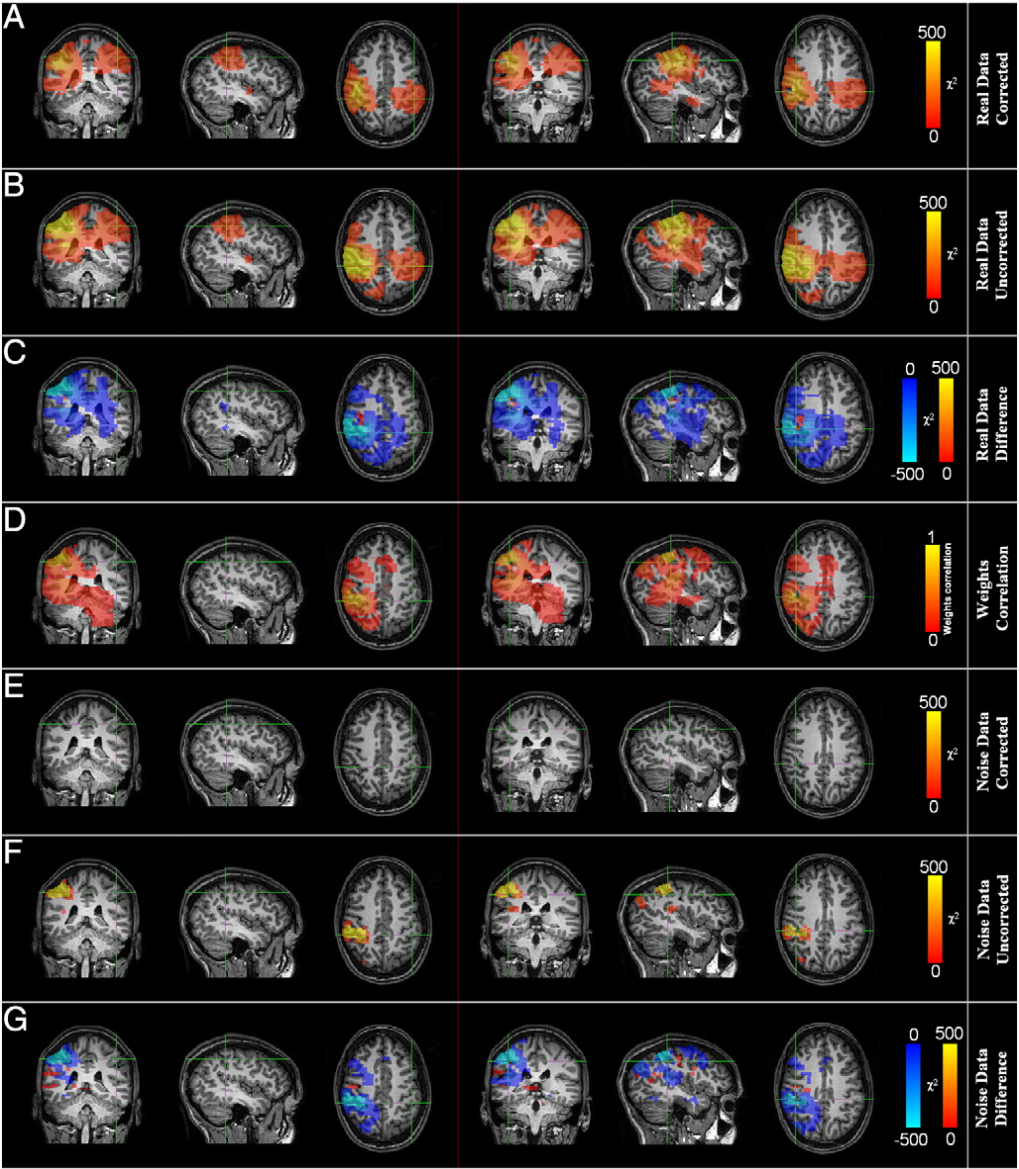
Resting state motor cortex connectivity delineated in the 4 Hz–80 Hz range in a single subject. A–D show the case for real data: A) Multivariate approach with correction for signal leakage thresholded at *α*_FWE_ < 0.05. B) The equivalent image without correction for signal leakage. C) Difference in *χ*^2^ between the corrected (A) and uncorrected (B) images. D) Beamformer weights correlation image. E–G show the case in which empty room noise data are projected through the same beamformer spatial filters as those derived from real data. E) Multivariate approach with correction (thresholded at *α*_FWE_ < 0.05; as expected no voxels were significant). F) Multivariate approach without correction (thresholded at *α*_FWE_ < 0.05). G) Difference in *χ*^2^ between corrected (E) and uncorrected (F) images.

Figs. 4A and B show resting state motor network connectivity maps, computed in a single subject, with the seed location in the right motor cortex. Here power–power modulations have been computed in the 4–80 Hz frequency range and results overlaid onto the subject's own anatomical MRI (*α*_FWE_ < 0.05). Fig. 4A shows the connectivity image with correction for signal leakage whereas Fig. 4B shows the equivalent case without correction for signal leakage. As expected, in both cases a seed in the right motor area highlights significant power–power coupling in the opposite hemisphere, specifically the left primary sensorimotor area. Fig. 4C shows the difference between the corrected and uncorrected case (i.e. the difference between Fig. 4A and Fig. 4B prior to thresholding). The effect of leakage correction is clear with areas defined as exhibiting significant connectivity prior to correction (Fig. 4B) eliminated when correction is applied (Fig. 4A). This is particularly apparent in the dorsolateral pre-frontal and posterior parietal cortices, and is highlighted by Fig. 4C.

Fig. 4D shows an image of the absolute Pearson correlation coefficient between beamformer weights at the seed and all other locations. This image has been thresholded at a Pearson correlation coefficient of 0.1. Correlation between beamformer weights necessarily induces a degree of correlation between projected time series (see Eq. (1)) and this image has been included to show the anisotropic nature of weights correlation with respect to the seed location. Spurious connectivity resulting from beamformer weights correlation will be reduced by signal leakage correction; note here that the brain regions where correction has most effect (Fig. 4C) also tend to exhibit high weights correlation. High connectivity within these areas has been effectively suppressed by the leakage correction, and thus does not appear as significant in Fig. 4A.

Figs. 4E–G show the multivariate technique applied in a case where empty room noise data have been projected through the same beamformer spatial filters as those derived from (and applied to) the real data. Here no-significant correlation should be observed and this is confirmed by Fig. 4E in which we show a connectivity image, based on noise data and corrected for linear interaction. Note that no voxel contained a *χ*^2^ statistic greater than the *α*_FWE_ < 0.05 threshold (*χ*^2^ = 144.8). Fig. 4F shows the case without correction; here the image has been thresholded (*α*_FWE_ < 0.05) and significant clusters appear close to the seed. For completeness, Fig. 4G shows the difference between the corrected (Fig. 4E) and uncorrected (Fig. 4F) images. Notice that the brain areas highlighted in this difference image (i.e. those areas most affected by correction) agree with those areas highlighted by the equivalent image derived using real data (Fig. 4C).

**Fig. 5:**
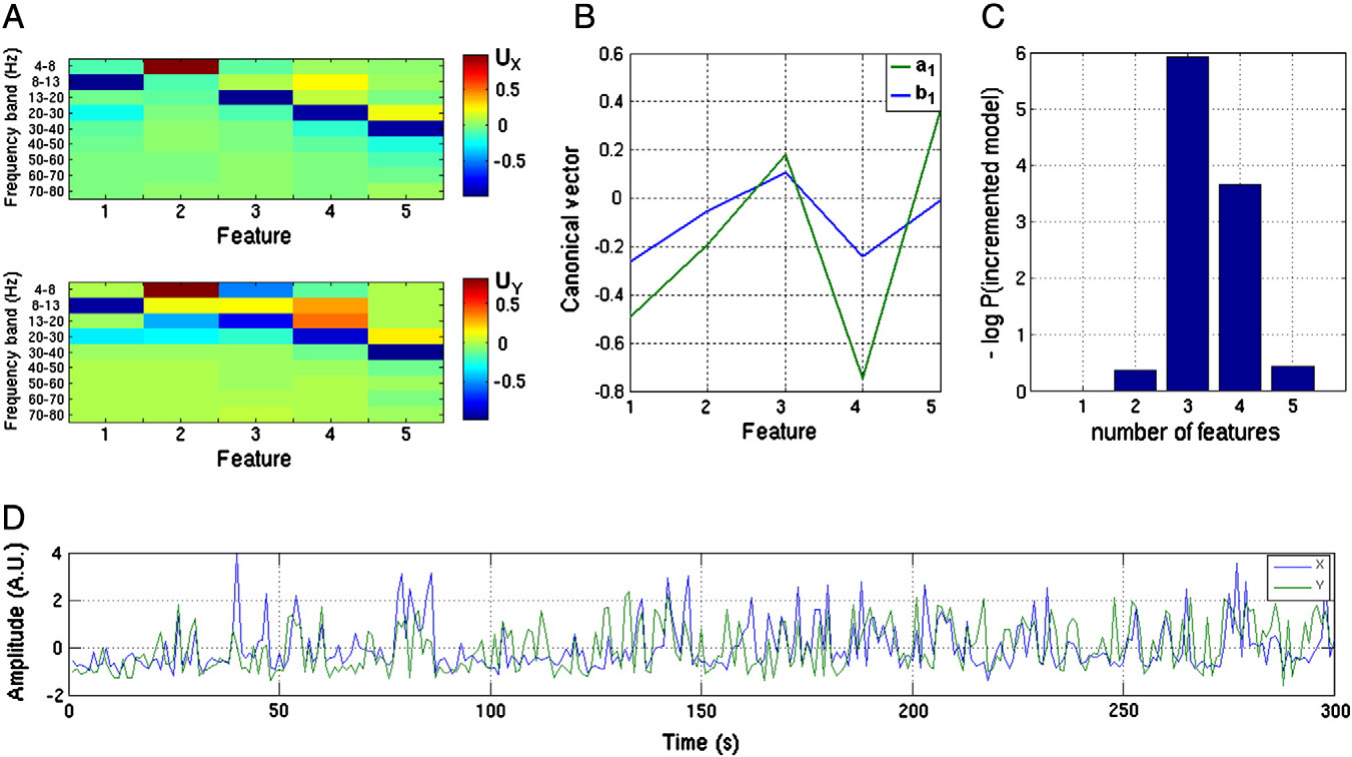
Assessing the contribution of the 5 features to connectivity between the left and right motor cortices for the first eigenmode. A) Contribution of each of the 9 frequency bands to the 5 orthogonal features (**U**_X_, upper panel, **U**_Y_ lower panel, colours represent magnitude of the elements of **U**_X_ and **U**_Y_). Note that the dominant mode (1) in both cases is a mixture of 8–13 Hz and 20–30 Hz power (mu rhythm). B) Canonical vectors **a**_1_ (green) and **b**_1_ (blue) from the first eigenmode showing the linear combinations of the features in Y and X respectively which maximally correlate. C) Bar chart showing the log probability that a model with *h* + 1 features improves on a model with *h* features. Values above 3 indicate that the more complex model is approximately twenty times more likely. Note that there is no evidence that including the second feature of X (predominantly 4–8 Hz power) improves the prediction of Y. D) Eigenmode timecourses for the first canonical variates **x**_1_′ (blue) and **y**_1_′ (green). The correlation (Pearson correlation 0.44) between these two linear mixtures of **X** and **Y** is known as the canonical correlation (Soto et al., 2010).

The images in Fig. 4A/B collapse the rich information content of projected MEG signals into a set of statistical quantities in order to visualise the spatial patterns of connectivity throughout the brain. However, having identified significant covariation observed in Fig. 4A it is important to return to the projected data in order to exploit the rich electrodynamic information available. Fig. 5 shows an example of how this might be possible using a classical statistical approach and **X** and **Y** selected from regions of interest based on the seed (in the right motor cortex) and the location of the local maximum in left motor cortex (Fig. 4A). We first tested whether there were any other eigenmodes of covariation demonstrating significant effects, using Eq. (14). For eigenmodes 1–5 we found significance levels of 9 ∗ 10^− 11^, 0.0047, 0.1622, 0.3846, 0.3128 respectively, suggesting that at this location there are two pairs of canonical variates (**x**_1_, **y**_1_, and **x**_2_, **y**_2_, Eqs. (16) and (17)) with significant linear correlation. For each significant mode we then hierarchically tested between models containing different numbers of features. The orthogonalisation matrices **U**_X_ and **U**_Y_ are shown in Fig. 5A; the features (columns) are organised in order of decreasing variance explained. For **X**, the dominant feature is power in the 8–13 Hz band (with some covariance in the 20–30 Hz band), the second feature consists of predominantly low frequency components (4–8 Hz). Fig. 5B shows the eigenvectors **a**_1_ and **b**_1_ corresponding to the most significant eigenmode. The next question to address is the relative importance of the different features (corresponding to elements of **a**_1_ and **b**_1_) to the correlation. In this case we test for data explained in **Y** and vary the number of features in **X**; we start with the feature explaining the most variance (leftmost column of **U**_X_) and test whether adding further features improves the model significantly. Fig. 5C shows the probability that there is no improvement between a model with *h* features and the previous model with *h* − 1 features. The scale of Fig. 5C is negative log so that values above 3 correspond to a significant improvement, with the more complex model (*h* features) being at least 20 times more likely than its predecessor (*h* − 1 features). It is clear from Fig. 5B that the addition of feature 2 (the 4–8 Hz band) makes almost no contribution to the model with a single feature, however, models incorporating features 3 and 4 (13–20 Hz and 20–30 Hz) improve significantly on those containing just 2 or 3 features respectively. At 5 features this improvement stops (we increased the total number of features to 10 to check that it did not rise again). The frequency bands highlighted here are in good agreement with those previously identified as playing a major role in motor cortex connectivity (Brookes et al., 2011a,b; Mantini et al., 2007); future work might use this technique to test hypotheses that a specific feature set, and corresponding frequency bands, has physiological relevance in connectivity between predefined brain locations. The tests on the second eigenmode identified the same significant features. In panel D we show the univariate projections, or canonical variates **x**′ and **y**′ that characterise the time-series of the interaction (over trials) of the first significant eigenmode.

## Discussion

We have applied a multivariate statistical framework to the study of functional connectivity in MEG data. More specifically, we have used the multivariate approach to assess power–power interactions between the timecourses of neural oscillations in multiple frequency bands, extracted from spatially separate brain regions. The approach presented offers a number of advantages over correlation methods previously used. 1) Interactions both within and across frequency bands can be assessed, and multiple couplings can be collapsed down to a single statistical image. 2) Correction for multiple comparisons across the image volume can be dealt with using a simple heuristic. 3) We present a method for addressing the leakage problem via a simple linear subtraction prior to multivariate analysis. We have shown the method to be viable in simulation, and that the false positive rate is well controlled, regardless of the imaging volume or the number of features examined. Finally, we have applied the technique to resting state MEG data and used it to identify cross hemisphere motor cortex interactions.

The biggest problem in MEG functional connectivity measurement is that of signal leakage between voxels in source space. This occurs as a result of the ill posed MEG inverse problem and ‘leakage’ in this context is a collective term for a number of separate effects including spatial spread of sources (characterised by the point spread function or resolution kernel) and spatial misattribution of sources due to, for example, inaccuracies in forward field computation. Previous work (Brookes et al., 2011a) has shown that signal leakage is both spatially wide spread and asymmetric with respect to the seed location and this is particularly problematic for connectivity measurement since spurious connectivity will necessarily result from leakage. An elegant approach to correct this was put forward by Soto et al. (2010) in which cross talk is eliminated by ignoring any regions in which the power–power coupling is symmetric (or the canonical vectors are co-linear). In this work we describe an alternative approach in which a univariate prediction of the measured signal at a test location is made, based on the signal at the seed location, and then subtracted.

The simulation results presented in Fig. 1 show clearly that this subtraction technique works effectively to remove blur around the seed location, whilst still enabling unbiased localisation of a spatially separate test source exhibiting a power–power coupling interaction with the seed. The effectiveness of the technique also extends to real data. In Fig. 4 the difference image (Fig. 4C) shows that whilst the major effect of correction is observed close to the seed location, effects also extend to distal areas of the cortex, and areas shown to exhibit significant connectivity in Fig. 4B (e.g. the dorsolateral pre-frontal and posterior parietal cortices) are effectively eliminated by the linear subtraction and do not appear significant in Fig. 4A. It is important to realise that the high degree of apparent connectivity between the seed and these other regions, prior to correction, can be explained (at least in part) by correlation between beamformer weights; this is evidenced by similarities between Figs. 4D and C. Correlation between weights introduces linear correlation between beamformer timecourses and such interactions would be expected to be removed by our correction. Interestingly, despite correction there remains a significant degree of high connectivity close to the seed; this effect was not observed in simulated data. It is possible that this reflects genuine physiological interaction (see discussion below) and represents an interesting topic for future work that could be readily probed using this method in conjunction with other, established, techniques such as phase lag metrics or imaginary coherence.

There are limitations to our leakage correction technique that should be discussed. We should point out that this technique does not solve the problem of source reconstruction and is highly dependent on the algorithm used to make the linear projection of channels into source space. For example, in this case the beamformer will produce linear weights which give better resolution of sources of higher power (which may not necessarily be those exhibiting the strongest connectivity). Similarly, it is important to realise that whilst this technique corrects for the effects of spatial leakage on the functional connectivity measured from the seed location to other brain regions, it does not correct for leakage from the test location to other brain areas. This could lead one to incorrectly infer the involvement of anisotropic (and possibly non-contiguous) spatially extended area of functional connectivity around the test location, which is due entirely to leakage from the test source. Note that this is essentially the same phenomenon as the spatial blurring that occurs in mapping changes in activity when using source reconstruction techniques. This effect could be tested by extending the technique described to inter-change the test and seed regions.

The leakage correction approach will not only remove zero lag correlation mediated by field spread, but also any genuine neurophysiological zero (or close to zero) lag correlation between the seed and target locations. This makes the approach conservative in terms of its sensitivity to true linear functional connectivity. It is possible that, in the case of true zero-lag physiological interactions, this could give a misleading picture of the relative amounts of connectivity present at different frequencies. Consider the case where there is a genuine and consistent neurophysiological zero lag interaction at 10 Hz between two sources. Any leakage correction based on a narrow band of frequencies (say 5–20 Hz) will make an erroneous overestimate of *β*_*UV*_; this will mean that at other frequencies (besides 10 Hz) apparent power couplings (due to uncorrected leakage effects) may result. For this to be a problem, non phase lagged coherence would have to persist for a significant fraction of the total time window of the data used. Similarly, the more frequency bands used, the smaller this bias will be. Existing evidence suggests that long range coherence is likely to be transient in nature (Friston, 1999; Rodriguez et al., 1999; Singer, 1999), and invasive studies have shown that stationary coherence domains for neural oscillations are of the order of 1 cm or less (Leopold et al., 2003). Indeed, in such (long-range) cases the basic assumptions underlying the beamformer spatial filter would also be violated. That said, this is an interesting topic for further investigation and could shed some light on the remaining seed blur observed with correction in Fig. 4A. Future work in this area could make use of the dual-core beamformer (Diwakar et al., 2011) or other inverse problem solutions not affected by correlated sources (Wipf et al., 2010; Zumer et al., 2007). Note also, that although such zero-lag physiological effects might skew the post-hoc analysis of the spectral profile of the interaction; they will not result in artefactual false positives in the mass-multivariate image.

It is well known that the SNR of MEG changes with frequency and is low for frequencies in the high γ band. This means that FC values computed using any technique will necessarily change with frequency and correlation or coherence values, computed between high gamma band signals, may be masked by poor SNR. This is a fundamental limitation of MEG that cannot be addressed by data processing. This limitation means that the multivariate technique presented here is biased towards frequency bands that exhibit high power; in the present case of resting state motor cortex connectivity, this means the α and β frequency bands. Although this problem is inherent to MEG recordings, it is possible that modifications to our technique might alter the bias. The simplest extension would be to normalise the columns of **X**_**P**_ and **Y**_**PR**_ to force them to have zero mean and unit norm. This would amplify the higher frequency components; it would cause marked changes to the orthogonalisation of **X**_**P**_ and **Y**_**PR**_ and also change the results presented in Fig. 5. However, this normalisation would also amplify high frequency noise which, for low degrees of freedom, would also bias results. For the present study we choose not to apply such normalisation, however it may help offset some frequency bias and future studies employing the multivariate method may consider this approach.

In the last section of the paper we performed a model comparison to assess which features provided useful information. We based this on a simple test of nested models, but this is not the only search strategy. We should note that this test was performed only at a single significant voxel, so that it would almost certainly not generalise to the brain volume. However, this specificity could potentially be useful. For example, different frequency–frequency mappings may correspond to mappings between different cortical areas; in which case one would begin with a proscribed univariate mapping on Y (e.g. sum of beta and alpha power) to make the algorithm less flexible but more spatially specific. Tests on this many to one mapping could also be carried out using existing Bayesian tools (Friston et al., 2008) which would also allow one to test between non-nested models.

In this demonstration we have used power exclusively, however the multivariate method can easily be generalised to other forms of interaction. Most simply, one can express **X** and **Y** using both their real and complex parts (rather than the absolute value); again removing zero-lag prediction of **X** on **Y** (over all frequency bands). This then becomes a method of identifying linear interactions between cortical areas (of non-zero lag). In the limit, for a single feature, this reduces to an equivalent form to those methods using only the imaginary part of the coherence (Guggisberg et al., 2008; Nolte et al., 2004). Similarly, it would be straightforward to use the phase of a low-frequency oscillation to predict power changes in other (higher frequency bands) as observed in Canolty et al. (2006) for example. There is no reason why these different covariates could not be appropriately orthogonalised and all used in the same analysis. A similar model comparison strategy could be used to test which models of interaction prevail; or indeed, it may well be that examination of the pertinent canonical variates reveals different time-courses for different forms of coupling.

Finally, the extension of the above methodology to group studies has yet to be thoroughly examined. The most straightforward method would be to develop univariate hypotheses on the form of coupling where the multivariate to univariate transform is made using the canonical vectors. These univariate tests could then be implemented with standard mass-univariate approaches. An alternative and promising approach which would preserve the multivariate nature of the test up to the group level would be to produce posterior probability maps based on different Bayesian models (Rosa et al., 2010).
